# Activation of Cryptic 3′ Splice-Sites by SRSF2 Contributes to Cassette Exon Skipping

**DOI:** 10.3390/cells8070696

**Published:** 2019-07-10

**Authors:** Heegyum Moon, Ha Na Jang, Yongchao Liu, Namjeong Choi, Jagyeong Oh, Jiyeon Ha, Xuexiu Zheng, Haihong Shen

**Affiliations:** School of Life Sciences, Gwangju Institute of Science and Technology, Gwangju 500-712, Korea

**Keywords:** SRSF2, cryptic 3′ splice-site, exon exclusion, intron retention, pre-mRNA splicing

## Abstract

Here we show that the serine/arginine rich splicing factor 2 (SRSF2) promotes cryptic 3′ splice-site (3′AG′) usage during cassette exon exclusion in survival of motor neuron (SMN2) minigenes. Deletion of the 3′AG′ (3′AG′1), its associated branch point (BP′) and polypyrimidine tract (PPT′) sequences directs SRSF2 to promote a second 3′AG′ (3′AG′2) with less conserved associated region for intron splicing. Furthermore, deletion of both 3′AG′1 and 3′AG′2 and their associated sequences triggered usage of a third 3′AG′3 that has very weak associated sequences. Interestingly, when intron splicing was directed to the 3′AG′ cryptic splice-sites, intron splicing from the canonical 3′AG splice-site was reduced along with a decrease in cassette exon inclusion. Moreover, multiple SRSF2 binding sites within the intron are responsible for 3′AG′ activation. We conclude that SRSF2 facilitates exon exclusion by activating a cryptic 3′AG′ and inhibiting downstream intron splicing.

## 1. Introduction

Splicing occurs at the consensus sequences near the 5′ and 3′ ends of introns, known as 5′ and 3′ splice-sites (5′ss and 3′ss) by a large, dynamic RNA-protein complex called the spliceosome [1]. In the spliceosome, small nuclear ribonucleoproteins (snRNPs) and several other proteins are recruited to the pre-mRNA [2]. The assembly of spliceosome, U1 small nuclear ribonucleoprotein (snRNP) is recruited to the 5′ss, U2 snRNP is directed to branch point (BP) sequence of intron. In the recognition of 3′ss and its associated region, U2 auxiliary factor 65 (U2AF65) identifies polypyrimidine tract (PPT), U2AF35 recognizes AG, SF1 recognizes BP sequence, U2 snRNA is basepaired with BP sequence to stabilize the spliceosome [1,3,4,5]. The recruitment of U4/U5/U6 tri-snRNP into spliceosome leads to catalytic activation of the spliceosome. Mutations in 5′ss, 3′ss, BP sequence and PPT causes genetic diseases by alter splicing efficiency [6]. Cryptic 5′ss or 3′ss instead of canonical splice-sites are sometimes activated [7]. In humans, while 5′ss, 3′ss and BP have become more degenerate and less conserved compared to lower eukaryotes such as yeast, polypyrimidine tract are stronger [8,9]. Alternative splicing generates diversity of mRNA isoforms and protein variants by selecting different combinations of 5′ and 3′ splice-site pairs to mediate gene regulation. High-throughput and genome-wide technologies indicate that alternative splicing occurs within transcripts from ~95% human multi-exon genes [10]. 5′ss, 3′ss, BP and PPT are not sufficient to select correct splice-site to regulate alternative splicing. In addition to these core splicing signals, RNA motifs known as exonic/intronic splicing enhancers or silences are required for further regulating alternative splicing. Therefore, more abundant enhancer and inhibitor sequences are identified within exon and introns in human genomes. The splicing enhancers or silencers are bound by trans-acting splicing factors such as serine/arginine-rich SR proteins [11]. SR proteins include N-terminal RNA recognition motif (RRM) that interacts with the pre-mRNA and C-terminal arginine/serine-rich (RS) protein interaction domain [12,13]. SRSF2 is a member of SR proteins, and first identified using a monoclonal antibody against purified spliceosome [14,15]. SRSF2 has been reported to interact with SSNG (S=C/G), G/A rich or purine-rich sequences from structure-based study, SELEX, CLIP-seq analysis [16,17]. In addition to the activator function in pre-mRNA splicing, SRSF2 was also reported to directly repress intron splicing [18]. In the alternative splicing, SRSF2 was demonstrated to either activate exon inclusion or exon skipping [19]. Furthermore, SRSF2 activates transcription elongation and couples transcription and splicing [20,21]. Spinal muscular atrophy (SMA) is a leading genetic cause of pediatric mortality, in which homozygous loss or mutation of survival motor neuron 1 (SMN1) gene occurred [22,23]. Humans also have a duplicate of SMN1 gene, SMN2, however, a mutation (C6T) in SMN2 causes predominant skipping of exon 7 to produce a truncated protein isoform that is degraded immediately [24]. An alternative cryptic exon in intron 6 of SMN1 and SMN2 was shown to be generated by exonization of an intronic Alu-like sequences of SMN1,2. [25,26]. HnRNP A1, SRSF1, SRSF2, SRSF10, TDP43, SRSF30c, PSF, U2AF65, hnRNP G and hnRNP M were shown to regulate exon 7 splicing in SMN2 [18,27,28,29,30,31,32,33,34]. SMN2 is present in all of the SMA patients but not able to compensate for the loss of SMN1 because of predominant exclusion of cassette exon 7 [27,28]. 29-*O*-(2-methoxyethyl) (MOE)-modified antisense oligonucleotides (ASOs) with phosphorotheioate backbone targeting splicing inhibitor was shown to rescues severe SMA mice [35].

In this manuscript, we demonstrate that SRSF2 significantly stimulates intron splicing at a cryptic splice-site (3′AG′1) with the most conserved associated region located at upstream of exon 7 in a SMN2 minigene. 3′AG′1 activation reduced canonical 3′AG activation and also decreased cassette exon inclusion. Interestingly, deletion of 3′AG′1 and its associated sequences, the BP (hereafter called BP′) and PPT (hereafter called PPT′) induced intron splicing at a second cryptic 3′ splice-site (3′AG′2) with less conserved associated region. Furthermore, deletion of both 3′AG′1 and 3′AG′2 induced activation of the third 3′AG′ (3′AG′3) activation that has the least conserved associated sequence. Importantly, multiple SRSF2 binding sites within the intron is responsible for 3′AG′ activation. Our results reveal that 3′AG′ activation and inhibition of canonical 3′AG splicing contribute to regulation of cassette exon skipping.

## 2. Materials and Methods

### 2.1. Plasmids Construction

E6-7, E6-7 (20, 112, 226, 334, 441) and E6-C was generated using the E6-8 minigene of SMN2 gene [18] as a template. The Δ3′AG′1, Δ3′AG′1/2, Δ3′AG′1/2/3, ΔSRSF2, E7/8ex, E6-5′cons, E7-ss-mut and SRSF2-△1, 2, 3, 4 constructs were generated by site-directed mutagenesis using the E6-8 minigene as a template. The E6-A2 construct was generated using the 5′ half of E6-8 construct and the 3′ half of adenovirus major late (AdML) construct [18] as templates and inserted into NheI, EcoRI, and XhoI restriction sites of pcDNA3.1 vector. SRSF2 expression plasmid was constructed with coding region of SRSF2 sequence into pcDNA6/myc/His A vector using BamHI and XhoI restriction sites. All primer sequences used to construct the minigenes are listed in Table S1.

### 2.2. Cell Culture, Transfection and RT-PCR

We grew 293T cells in Dulbecco’s Modified Eagle Medium (DMEM) media supplemented with antibiotics and 10% FBS at 37 °C and in 5% CO2. SMA patient cell line GM3813 was cultured in DMEM media with DMEM and 10% FBS by adding non-essential amino acids. We transfected all of the minigene plasmids into cells using polyethyleneimide (PEI) as previously described [18]. Total cellular RNA was extracted using the RiboEx reagent (GeneAll) and conventional RT-PCR was performed as described previously [18]. All primer sequences are listed in Table S1. Quantitation of RT-PCR result was performed by ImageJ software as described previously [29].

### 2.3. Knockdown of SRSF2 with Lentivirus-Mediated shRNA

The SRSF2 targeted shRNA lentivirus was generated by co-transfection of SRSF2-targeting shRNA plasmid (openbiosystems) and helper plasmids into 293T cells using polyethyleneimide (PEI) reagent. Lentivirus containing supernatant was harvested and added to the GM3813 cells with the supplement of 10 μg/mL polybrene.

## 3. Results

### 3.1. SRSF2 Promotes Cryptic 3′AG′ Activation 

**Figure 1:**
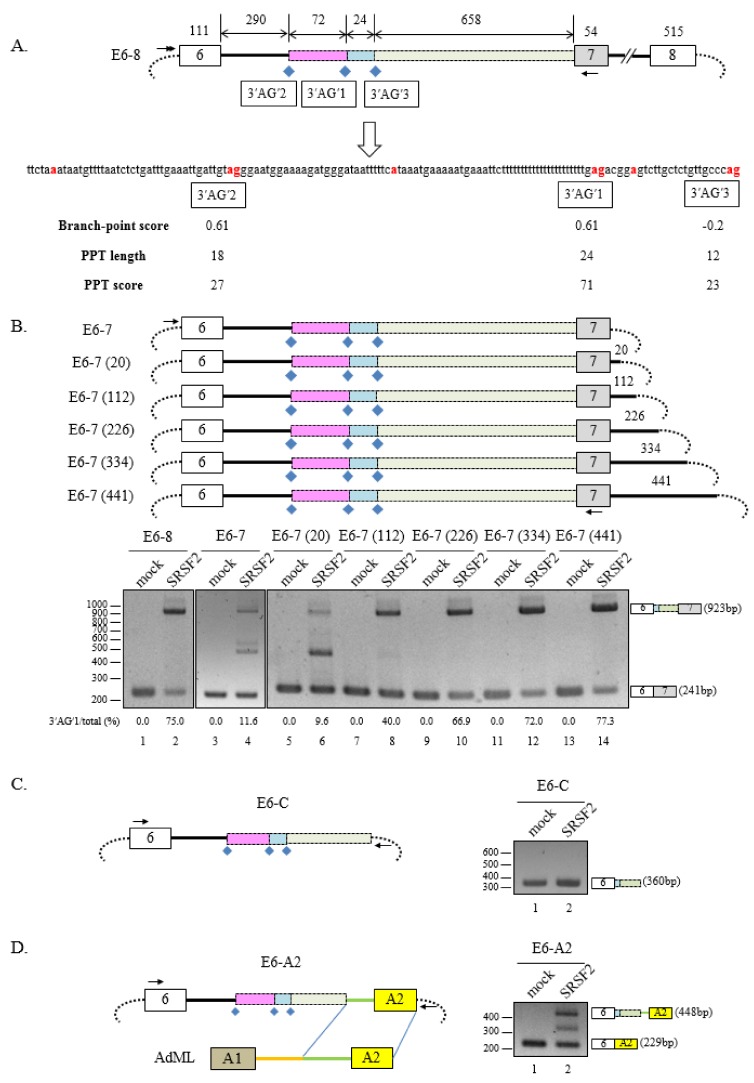
The serine/arginine rich splicing factor 2 (SRSF2) promotes cryptic 3′AG′ splice-site activation. (**A**) Schematic diagram of the E6-8 minigene. Exons are depicted as numbered gray and white boxes, introns as solid lines, vector sequence as dots and acryptic 3′AG′ sites as blue diamonds. The 3′AG′1, 3′AG′2 3′AG′3 and associated sequences are shown below the schematic diagram. The fragment from 3′AG′3 to the end of intron 6 is indicated in light green, the fragment from 3′AG′1 to 3′AG′3 is indicated in cyan, and the fragment between 3′AG′2 and 3′AG′1 is indicated in pink. Additionally, the 3′AG′ sequence and BP′ adenosine are highlighted in red, the primer binding sites used for RT-PCR reaction are indicated as arrows, and BP scores, PPT length, and PPT scores of each 3′AG′s are shown. (**B**) (Upper panel) Schematic diagram of the E6-7 minigene and various constructs. The primer bindings sites used for RT-PCR analysis are indicated as arrows. (Lower panel) RT-PCR analysis of intron 6 splicing within the E6-8, E6-7 minigene or various mutant constructs using RNAs extracted fromSRSF2-expressing or pcDNA transfected cells. The sizes of products are shown at the right of all figures here and below. (**C**) (Left panel) Schematic diagram of the E6-C minigene indicating primer binding sites. (Right panel) RT-PCR analysis of the E6-C minigene using RNAs extracted from SRSF2-expressing or pcDNA transfected cells. (**D**) (Left panel) Schematic diagram of the E6-A2 and AdML minigenes indicating primer binding sites. (Right panel) RT-PCR analysis of intron splicing within the E6-A2 minigene in pcDNA- or SRSF2-expressing cells.

We have previously demonstrated that SRSF2 promotes a cryptic 3′ splice-site (3′AG′) that is located at 682 nt upstream of 3′AG at exon 7 while simultaneously suppressing splicing at the 3′AG splice-site in the SMN2 minigene [18]. In the minigene we studied, the cryptic exon that was previously described [25,26] was not included. To differentiate 3′AG′ from other putative cryptic splice-sites that are described in the other parts of the manuscript, we designated the 3′AG′ as 3′AG′1, a 22 nt polyuridine sequence that functions potentially as a polypyrimidine tract as PPT′, and a potential branch-point sequence upstream of the PPT as BP′ (summarized in Figure 1A). 

An important question was whether remote exons and introns are required for SRSF2-mediated cryptic 3′AG′ splice-site activation. To address the question, we constructed the E6-7 minigene (Figure 1B, upper panel) where intron 7 and exon 8 were deleted from the E6-8 minigene, which includes shorter length (1 kb) of intron 6 and full length of exon 8 [31]. As previously reported, E6-8 minigene produced the cryptic 3′AG′1 splice-site activated product (Figure 1B, lower panel, lane 2) [18]. Similarly, E6-7 minigene also produced 3′AG′1 activated product but to a much lesser extent (Figure 1B, lower panel, lane 4) compared to the E6-8 minigene (lane 2). Additionally, splicing at the canonical 3′AG splice-site was suppressed, but not as extensively as seen in the E6-8 minigene (Figure 1B, lower panel, lane 4), indicating that downstream introns and exons are necessary for SRSF2 activity. It was possible that the length of the downstream intron could also affect SRSF2 function. To address this possibility, we constructed a series of minigenes where 20 [E6-7 (20)], 112 [E6-7 (112)], 226 [E6-7 (226)], 334 [E6-7 (334)], and 441 [E6-7 (441)] nucleotides of intron 7 were retained, respectively (Figure 1B, upper panel). We found that SRSF2 stimulated splicing at 3′AG′1 in the E6-7 (20) minigene to similar levels as in the E6-7 minigene (Figure 1B, lane 6). These results reveal that the 20 nt intronic sequence is not important for intron splicing at the 3′AG′ cryptic splice-acceptor sites. Additionally, for SRSF2-expressing cells, more splicing at 3′AG′1 occurred in longer intronic sequences than do shorter ones (Figure 1B, lanes 8, 10, 12 and 14). Therefore, 3′AG′ activation occurs more readily with longer downstream introns in the presence of SRSF2 in SMN pre-mRNA. Finally, it was important to determine whether splicing occurs at cryptic splice-sites in a gene construct with only one exon and intron. Surprisingly, splicing at 3′AG′1 occurs in a minigene lacking exon 7 (E6-C) (Figure 1C, left panel) even if SRSF2 is absent (Figure 1C, right panel, lane 1). That cryptic splice-sites are more easily activated in minigenes lacking an exon was confirmed by constructing a chimeric minigene (E6-A2) composed of a part from exon 6 and upstream intron 6 sequences in the E6-7 minigene and the other part from exon 2 (A2) and upstream intron of the adenovirus major late (AdML) pre-mRNA derivative (Figure 1D, left panel). Splicing of cryptic exon was observed only when SRSF2 was expressed (Figure 1D, right panel, compare lanes 1 and 2). Therefore, we conclude that SRSF2 suppresses intron 6 splicing by activating cryptic 3′AG′. To consider the possibility that reduced SRSF2 expression regulates cryptic 3′AG′ activation, we performed shRNA-mediated reduction of SRSF2. As shown in Supplementary Figure S1, the cryptic 3′AG′ splice-site was not activated by SRSF2-targeting shRNA (Supplementary Figure S1).

### 3.2. Deletion of 3′AG′ Region Induces SRSF2-Dependent Usage of Alternate 3′AG′

**Figure 2:**
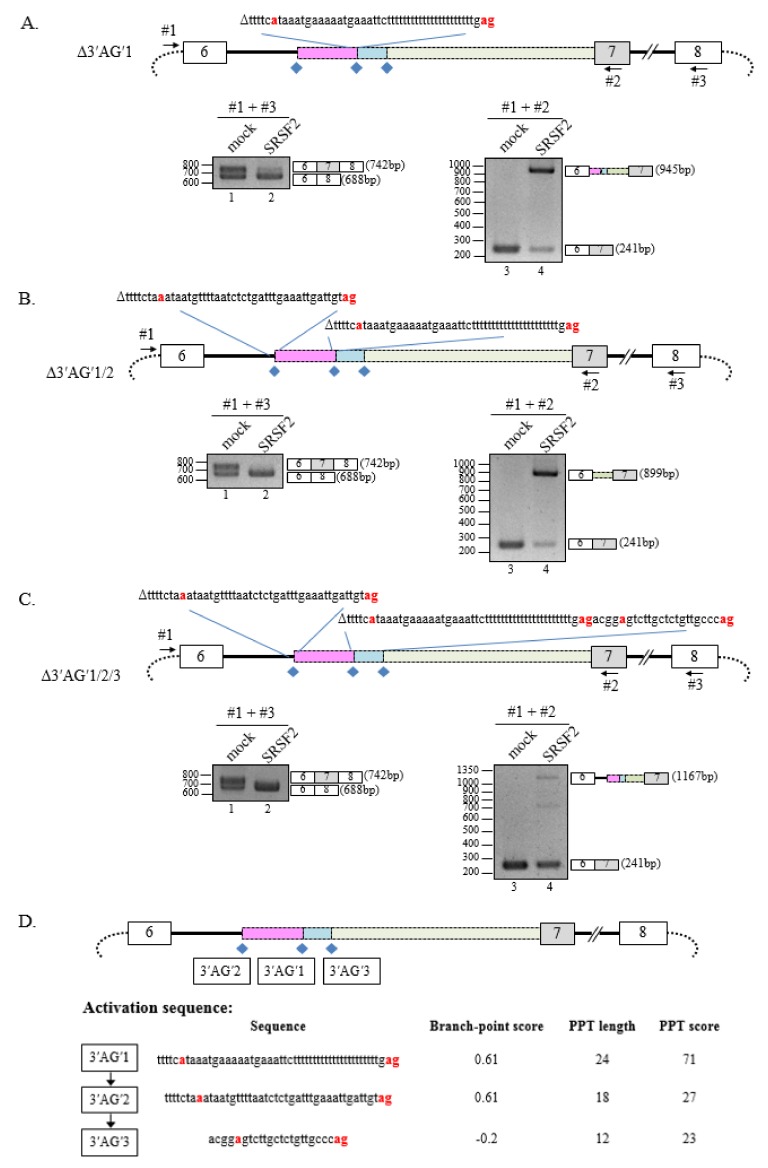
Deletion of 3′AG′1 region induces SRSF2-dependent usage of alternate 3′AG′. (**A**) (Upper panel) Schematic diagram of the Δ3′AG′1 minigene. The nucleotide deletion is indicated at top but is otherwise the same as Figure 1A. (Lower panel) RT-PCR analysis of the Δ3′AG′1 minigene using RNA extracted from pcDNA- or SRSF2-expressing cells with primer pairs #1 and #3 (left) or #1 and #2 (right). (**B**) (Upper panel) Schematic diagram of the Δ3′AG′1/2 minigene. Both nucleotide deletions are indicated at top but is otherwise the same as Figure 1A. (Lower panel) RT-PCR analysis of the Δ3′AG′1/2 minigene using RNA extracted from pcDNA- or SRSF2-expressing cells with primer pairs #1 and #3 (left) or #1 and #2 (right). (**C**) (Upper panel) Schematic diagram of the Δ3′AG′1/2/3 minigene. Nucleotide deletions encompassing all three cryptic splice-sites are indicated at top but is otherwise the same as in Figure 1A. (Lower panel) RT-PCR analysis of the Δ3′AG′1/2/3 minigene using primers #1 and #3 (left) or #1 and #2 (right). (**D**) Summary of the order of activation (splice-site usage) of the various cryptic splice-sites based on BP score and PPT strength.

Although there are several putative cryptic splice-sites within the SMN minigene (Figure 1A), it was unknown whether deletion of 3′AG′1 would prevent SRSF2 from influencing intron 6 splicing. To test this possibility, the Δ3′AG′1 minigene was generated with deletions of 3′AG′1, BP′1, and PPT′1 (Figure 2A). Cells expressing both SRSF2 and the Δ3′AG′1 minigene favored cassette exon exclusion (Figure 2A, lower panel, lane 2). Interestingly, another cryptic 3′ss (3′AG′2) located 72 nt upstream of 3′AG′1 was activated by SRSF2 (Figure 2A, lower panel, lane 4). The 3′AG′2 region includes a much weaker and shorter PPT compared with 3′AG′1 (Figure 1A) allowing for the better conserved 3′AG′1 to be activated in advance by SRSF2.

However, in the absence of a strong cryptic splice-site, alternative cryptic 3′ss splicing using a less conserved, weaker 3′AG′ might be possible when SRSF2 is present. These results raised the possibility that yet another cryptic splice-site, in the absence of both 3′AG′1 and 3′AG′2, could mediate alternative splicing of the SMN minigene. Therefore, two other minigenes with deletions of two or three cryptic splice-sites, Δ3′AG′1/2 and Δ3′AG′1/2/3, respectively, were generated to test for alternative splicing in SRSF2-expressing cells (Figure 2B,C, upper panel). As before, cassette exon exclusion was favored by both Δ3′AG′1/2 and Δ3′AG′1/2/3 minigenes (Figure 2B,C, lower panel, lane 2). However, when testing for splicing of intron 6, deletions of both 3′AG′1 and 3′AG′2 promoted alternative splicing from a third cryptic splice-site, 3′AG′3, only in cells overexpressing SRSF2 (Figure 2B, lower panel, lane 4). This was interesting because 3′AG′3 is located 24 nt downstream of 3′AG′1 and contains a much shorter PPT sequence and has less well-conserved BP sequences than 3′AG′2 (Figure 1A). Moreover, the absence of all three cryptic splice-sites (3′AG′1, 3′AG′2 and 3′AG′3) in the Δ3′AG′1/2/3 minigene resulted in only or mostly canonically spliced exons in the non-transfected or SRSF2-expressing cells, respectively (Figure 2C, lower panel, lane 4). The results imply that SRSF2 promotes usage of most conserved cryptic 3′AG′ based on branch-point score, PPT length, and PPT (SVM-BPfinder, http://regulatorygenomics.upf.edu/Software/SVM_BP/), summarized in Figure 2D.

### 3.3. Multiple SRSF2-Binding Sequences and Clusters Are Necessary for 3′AG′ Activation by SRSF2

**Figure 3:**
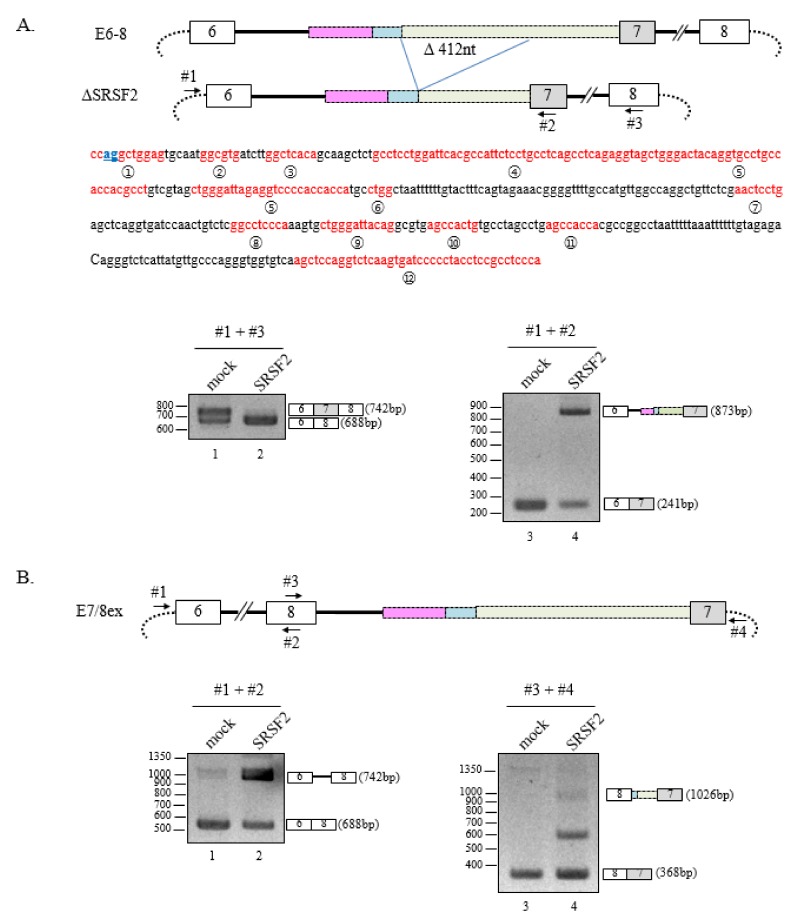
Multi SRSF2-binding sequences and clusters are responsible for 3′AG′ activation by SRSF2. (**A**) (Upper panel) Schematic diagram of the ΔSRSF2 minigene. The nucleotide deletion is indicated using blue lines. The primer binding sites used in lower panels are indicated with arrows. (Middle panel) The sequence of deleted 412 nt is shown. Potential SRSF2 binding sites and clusters are shown in red and numbered. (Lower panel) RT-PCR analysis of the ΔSRSF2 minigene in SRSF2-expressing cells using primers #1 and #3 (left) or #1 and #2 (right). (**B**) (Upper panel) Schematic diagram of the E7/8ex minigene where a portion including intron 6 though exon 7 was swapped with a region containing intron 7 through exon 8. The primer binding sites used in lower panels are indicated with arrows. (Lower panel) RT-PCR analysis of the E7/8ex minigene in SRSF2-expressing cells using primers #1 and #2 (left) or #3 and #4 (right).

Because SRSF2 is an RNA-binding protein with degenerate sequence-specificity (unlike other SR proteins), the pre-mRNA usually includes many potential SRSF2 binding sequences [19]. We observed that 12 potential SRSF2 binding sequences or clusters are located downstream of 3′AG′1 (Figure 3A, middle panel). In contrast, only one potential SRSF2 binding site is located around the canonical 3′AG. Therefore, it was possible that the abundance of potential SRSF2-binding sequences downstream of 3′AG′1 were responsible for activation of cryptic splicing. To address this possibility, we deleted 412 nucleotides from the E6-8 minigene to remove potential SRSF2 binding sites (ΔSRSF2, Figure 3A, upper panel). Although SRSF2 mediated exon skipping as before (Figure 3A, lower panel, lane 2), we also observed mostly the unspliced isoform (873bp) but no cryptically spliced isoforms of the ΔSRSF2 minigene (Figure 3A, lower panel, lane 4). These data provide strong evidence that the region of the E6-8 minigene encompassing the 412 nucleotides deletion is important for SRSF2 function in cryptic 3′AG′ activation. To further narrow the binding target sequences of SRSF2, we generated several deletion mutants of the ΔSRSF2 minigene with 103, 32, 26 or 40 nucleotide deletions (SRSF2-Δ1, SRSF2-Δ2, SRSF2-Δ3 and SRSF2-Δ4, respectively, Supplementary Figure S2A). With these smaller deletions, SRSF2 was still able to activate cryptic splicing at 3′AG′1 in all the mutant constructs (Supplementary Figure S2B). Therefore, it appears likely that several of these SRSF2-binding sequences together are responsible for cryptic splice-site activation by SRSF2. Our next task was to determine whether 3′AG′ could still be activated by SRSF2 if intron 6 is located downstream of the cassette exon (exon 7). Therefore, we swapped intron 6/exon 7 with intron 7/exon 8 (E7/8ex, Figure 3B, upper panel) and found that while intron 7 splicing was inhibited (Figure 3B, lower panel, lane 2), cleavage at 3′AG′1 was still possible (Figure 3B, lower panel, lane 4). Therefore, we concluded that 3′AG′ activation is not due to the location of the intron containing the cryptic splice-site.

### 3.4. 5′Splice-Site Mutations do not Affect SRSF2-Mediated 3′AG′ Activation

**Figure 4:**
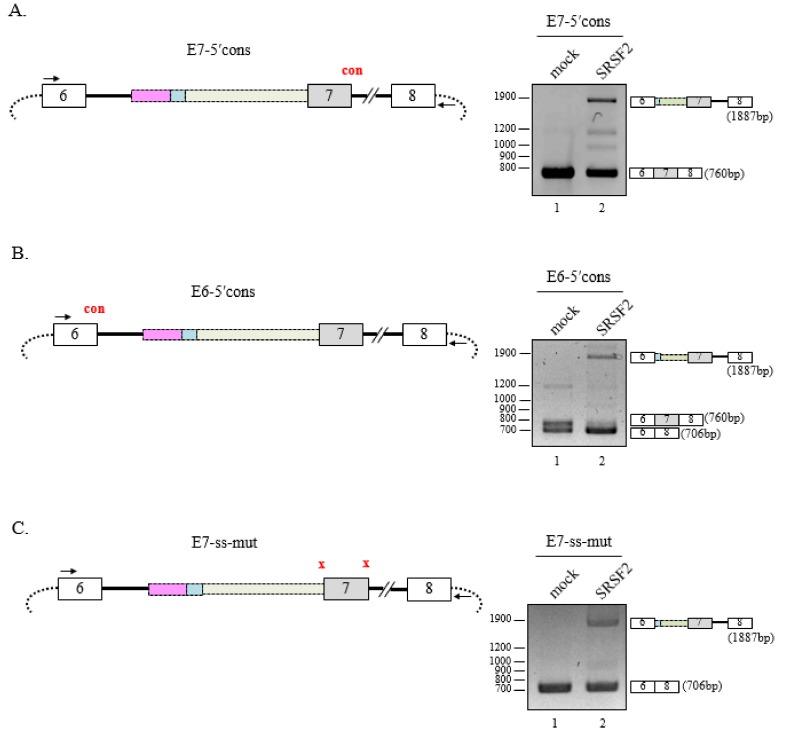
5′ splice-site mutations do not affect SRSF2-mediated 3′AG′ activation. (**A**) (Left panel) Schematic diagram of the E7-5′cons minigene where the 5′ss has been changed to a conserved sequence. (Right panel) RT-PCR analysis of the E7-5′cons minigene in SRSF2-expressing cells. (**B**) (Left panel) Schematic diagram of the E6-5′cons minigene including the conserved 5′ss sequence of exon 6. (Right panel) RT-PCR analysis of the E6-5′cons minigene in SRSF2-expressing cells. (**C**) (Left panel) Schematic diagram of the E7-ss-mut minigene where the splice signals of exon 7 have been abolished. (Right panel) RT-PCR analysis of the E7-ss-mut minigene in SRSF2-expressing cells.

It remained unknown whether 5′ splice-site mutations affect cryptic 3′ splice-site activation by SRSF2. First, we tested the E7-5′cons minigene where the 5′ss of the cassette exon (exon 7) was mutated to a more well-conserved sequence (Figure 4A, left panel). Although cassette exon exclusion was not observed, splicing at 3′AG′1 was observed when SRSF2 was overexpressed (Figure 4A, right panel). Therefore, a better conserved 5′ss did not affect SRSF2-mediated cryptic splicing. Next, we examined the E6-5′cons minigene where the 5′ss of exon 6 was mutated to a conserved sequence (Figure 4B, left panel). As before, alternative splicing of the minigene at 3′AG′ was observed but both cells, overexpressing SRSF2 or not, were now able to activate 3′AG′1 (Figure 4B, right panel). Finally, we wondered whether disruption of 5′ splice-donor and 3′ splice-acceptor site in the cassette exon would affect SRSF2-mediated 3′AG′ activation. We tested another mutant minigene, E7-ss-mut, where the splice-site sequences of exon 7 were abolished, effectively converting E6-8 to a two exon minigene (Figure 4C, left panel). Here, alternative splicing at 3′AG′ was still possible in the absence of splicing signals but the majority of transcripts had excluded exon 7 (Figure 4C, right panel, lane 2). Together, these results provide strong evidence that 3′AG′ can always be activated by SRSF2 regardless of 5′ss strength.

## 4. Discussion

In this study, our data showed that among three cryptic 3′AGs, the 3′AG′1 with most conserved associated sequence of intron 6 is first activated by SRSF2 under conditions favoring exon skipping, and 3′AG′ activation reduced splicing from the canonical 3′AG to inhibit cassette exon splicing. Interestingly, when we deleted 3′AG′1 and its upstream PPT′ and BP′, we found that the second 3′AG′2 having less conserved associated region among two remaining 3′AG′s was activated. Furthermore, deletion of both 3′AG′ (3′AG′1, 3′AG′2) induced the activation of the third remaining 3′AG′3 by SRSF2. Importantly, 3′AG′ activation requires multiple SRSF2 binding sites within the intron. It was previously reported that a cryptic exon in intron 6 is included in a mRNA to produce another isoform of SMN. Splicing of the cryptic exon occurred by an exonization event of Alu element [25,26]. Here we identified novel three cryptic 3′AG′ in intron 6 of SMN2 gene, that was activated by SRSF2 in a SMN2 minigene.

We previously reported that SRSF2 directly inhibits intron 7 splicing and BP surrounding 10 nt is essential for the SRSF2-mediated intron repression, and the intron 7 inhibition contribute directly to cassette exon exclusion [18]. In this manuscript, we present that SRSF2 suppresses another flanking intron 7 splicing by promoting a cryptic 3′AG′ splice-site. The previous and current results demonstrate that SRSF2 simultaneously inhibits flanking 5′ss and 3′ss splicing by either directly inhibiting downstream intron splicing or activating cryptic 3′AG′. These observations provide an insight for the regulation of SR proteins.

Although 3′AG′ activation by other SR proteins, such as SRSF1 and SRSF5, was described previously [36], 3′AG′ activation by SRSF2 has never been reported. An important observation is that SRSF2 could activate the 3′AG′3 with much less conserved associated BP′ and PPT′. Among three potential 3′AG′s, the 3′AG′ with most strong associated sequences, including conserved BP′ and abundance of pyrimidine nucleotides in PPT′, are always activated. 3′AG′ activation did not occur in a location-dependent manner. Notably, deletion of all 3′AG′s did not totally abrogate SRSF2 function as exon skipping was still possible. It is possible that the presence of many SRSF2-binding sequences could still bring the splicing machinery around to the 3′AG′s thus deletion of 3′AG′s reduced canonical 3′AG splicing. Others have suggested that SR proteins interact with RNA in one location to activate splicing. However, it was also reported that SR proteins coordinate with each other to activate splicing [37,38]. In agreement with these reports, we demonstrated that multiple SRSF2-binding sites are required for 3′AG′ activation.

Compared with other SR proteins, SRSF2 has more diverse binding consensus [16]. SSNG (S = C/G), GA-rich or purine-rich sequences were identified as the consensus sequences in different approaches [16,17]. Although we performed shRNA treatment for SRSF2, we could not observe endogenous 3′AG′ activation by reduced SRSF2 expression. The difference of the results is also caused by experimental differences: minigenes or endogenous pre-mRNA, overexpression or shRNA treatment. Another possibility is that the minigene we tested include only ~1 kb sequence, but the other ~4.8 kb RNA has important roles in the endogenous splicing. Furthermore, additional regulatory mechanisms play roles in the endogenous 3′AG′. In addition, we performed all experiments with SRSF2-overexpressing cells, and the shRNA effect is not always opposite to that of overexpression. The degenerate consensus of SRSF2 binding targets probably make endogenous SRSF2 forms a multi-molecular complex with different roles. SRSF2 mutations occur frequently in patients with myelodysplastic syndromes (MDS) [39,40]. How these mutations in SRSF2 regulate 3′AG′ in MDS still needs to be determined.

## Supplementary Materials

The following are available online at https://www.mdpi.com/2073-4409/8/7/696/s1, Figure S1: SRSF2 did not affect endogenous 3′AG′ activation. Figure S2: Serial deletions of putative SRSF2 binding sites did not disrupt 3′AG′ activation by SRSF2. Table S1: Primer list.
