# Supplementary material for: Activation of Cryptic 3′ Splice-Sites by SRSF2 Contributes to Cassette Exon Skipping
Source: Cells. 2019 Jul 10;8(7):696. doi: 10.3390/cells8070696 (PMC6678912; doi:10.3390/cells8070696)
Supplement: Supplementary file 1 [file cells-08-00696-s001.pdf]

cells

MDPI

Supplementary Materials: Activation of Cryptic 3′ Splice-Sites by SRSF2 Contributes to Cassette Exon Skipping

Heegyum Moon, Ha Na Jang, Yongchao Liu, Namjeong Choi, Jagyeong Oh, Jiyeon Ha, Xuexiu Zheng and Haihong Shen

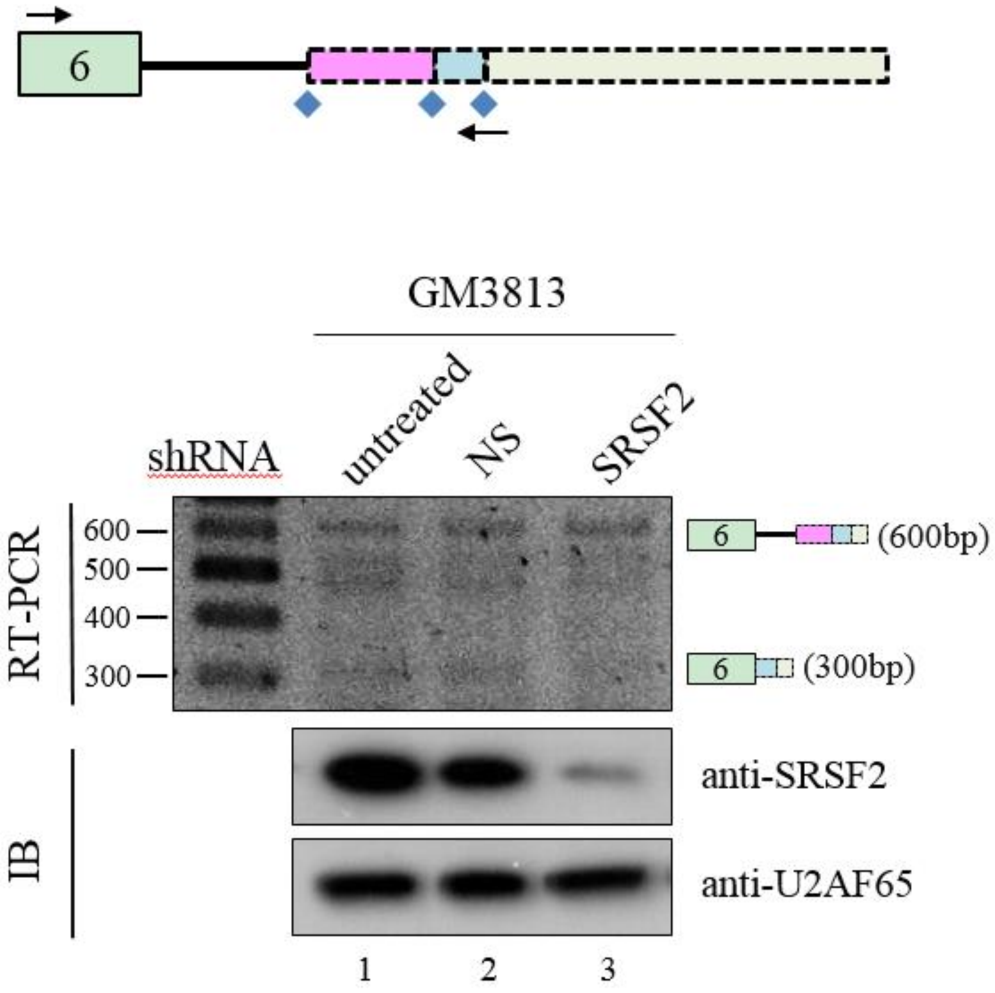

Figure S1. SRSF2 did not affect endogenous 3′AG′ activation. (Upper panel) Primers for 3′AG′ activation are shown with arrows. (Lower panel) RT-PCR analysis to 3′AG′ activation in Spinal Muscular Atrophy patient cells (GM3813) treated with non-silencing and SRSF2-targeting shRNA. Immunoblotting with anti-SRSF2 for SRSF2 expression is shown below, U2AF65 expression is shown as a control.

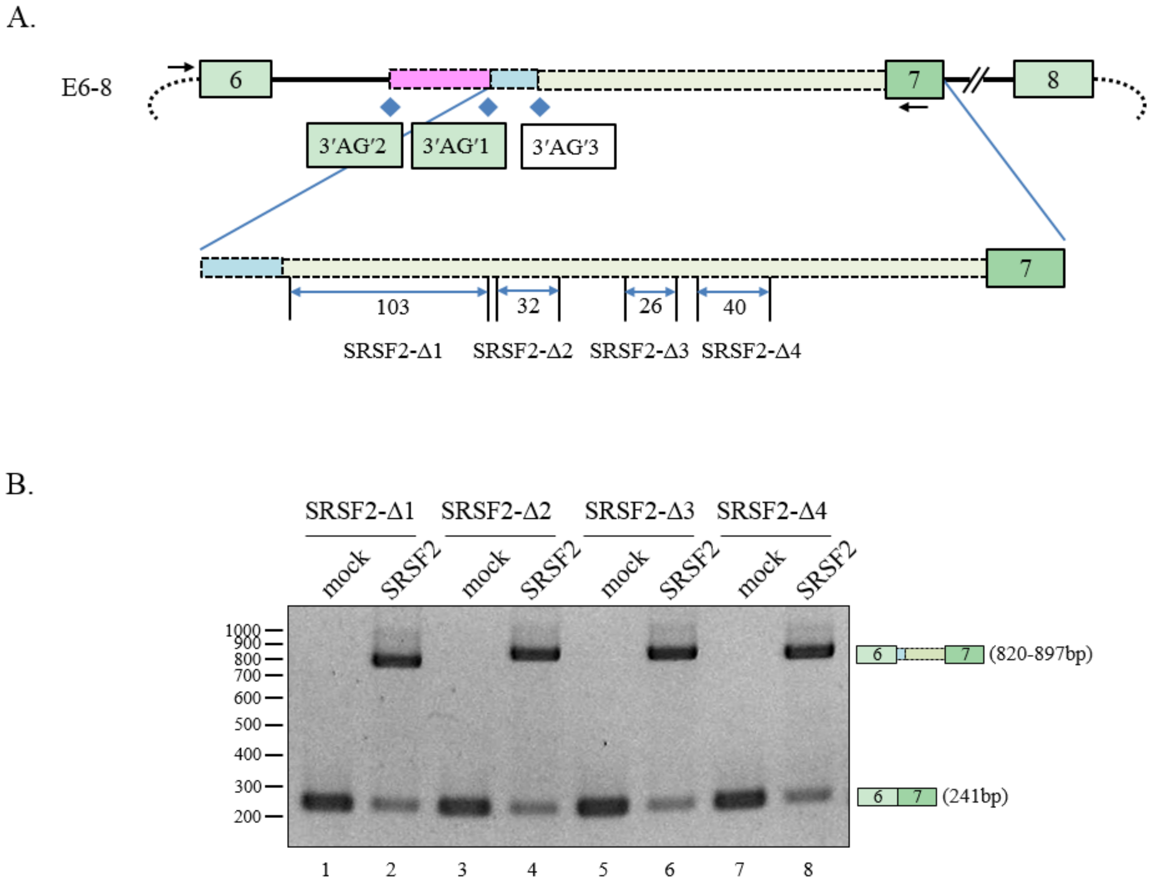

Figure S2. Serial deletions of putative SRSF2 binding sites did not disrupt 3′AG′ activation by SRSF2. (A) Locations of the deleted fragments in the pre-mRNA are indicated. (B) RT-PCR analysis to detect intron6 splicing in cells overexpressing SRSF2 or transfected with pcDNA.

Table 1. Primer list.

**PLASMID CONSTRUCTION**	
**NAME**	**SEQUENCE**
SMN.E6(B).F	TACTCGGATCCATAATTCCCCCACCACCTCC
SMN.E8(X).R	CTAACCTCGAGAACAGTACAATGAACAGCCATG
SMN.E7.R	CGATGCTCGAGTCCTTAATTTAAGGAATGTGAG
SMN.E7(I7-20).R	CGGTGCTCGAGCATAATGCTGGCAGACTTACTC
SMN.E7(I7-112).R	CCTAGCTCGAGACCTTTCAACTTTTTAACATCTG
SMN.E7(I7-226).R	CATAGCTCGAGGTTTCTTCCACATAACCAACC
SMN.E7(I7-334).R	AATCGCTCGAGTGCAGTATGCCTAGGTTATCC
SMN.E7(I7-441).R	CCACACTCGAGGCAAATGAGAAATTAGAACCAG
Δ3’AG’1.F	GAAAAGATGGGATAATACGGAGTCTTGCTCTG
Δ3’AG’1.R	CAGAGCAAGACTCCGTATTATCCCATCTTTTC

Δ3'AG'1/2.F	CTTTTGAATTGAAATTATACTTTGGAATGGAAAAGATGGGATAA
Δ3'AG'1/2.R	TTATCCCATCTTTTCCATTCCAAAGTATAATTTCAATTCAAAAG
Δ3'AG'1/2/3.F	GGAATGGAAAAGATGGGATAATGCTGGAGTGCAATGGCGTG
Δ3'AG'1/2/3.R	CACGCCATTGCACTCCAGCATTATCCCATCTTTTCCATTCC
E7-ss-mut1.F	AAATTAAGGAAAAAGTCTGCCA
E7-ss-mut1.R	TGGCAGACTTTTTCCTTAATTT
E7-ss-mut2.F	TTTTCCTTACCCGGTTTTAGAC
E7-ss-mut2.R	GTCTAAAACCGGGTAAGGAAAA
E7/8ex-1.R	CACATAACTACAAAAAAATTG
E7/8ex-2.F	TTTTGTAGTTATGTGGGTTTGTGGAAAACAA
E7/8ex-3.R	GACATGGCTGTTCATTGTACTG
E7/8ex-4.F	CTGTTCATTGTACTGGTAAGTCTGCCAGCATT
E7/8ex-5.R	ATAAAGTTTTACAAAAGTAAG
E7/8ex-6.F	CTTTTGTAAAACTTTATACTTTGTTTTGTAAATT
E6-5'cons.F	ATACTGGCTATTATCAGGTAAGTAATCACTC
E6-5'cons.R	GAGTGATTACTTACCTGATAATAGCCAGTAT
ΔSRSF2.F	ACGGAGTCTTGCTCTGTTGCAAGTTGTGGGATTGTAGGCA
ΔSRSF2.R	TGCCTACAATCCCACAACTTGCAACAGAGCAAGACTCCGT
SRSF2-Δ1.F	CCCAGGCTGGAGTGCAATGTCGTAGCTGGGATTAGAG
SRSF2-Δ1.R	CTCTAATCCCAGCTACGACATTGCACTCCAGCCTGGG
SRSF2-Δ2.F	CCACCACGCCTGTCGTAGCTAATTTTTTGTACTTTCAGTAG
SRSF2-Δ2.R	CTACTGAAAGTACAAAAAATTAGCTACGACAGGCGTGGTGG
SRSF2-Δ3.F	CAGGTGATCCAACTGTCTCGCGTGAGCCACTGTGCCT
SRSF2-Δ3.R	AGGCACAGTGGCTCACGCGAGACAGTTGGATCACCTG
SRSF2-Δ4.F	GTTGCCCAGGGTGGTGTCAAAGTTGTGGGATTGTAGGCA
SRSF2-Δ4.R	TGCCTACAATCCCACAACTTTGACACCACCCTGGGCAAC
E6-A2(1).F	TACTCGCTAGCATAATTCCCCCACCACCTCC
E6-A2(1).R	CTAACGAATTCCCTCTAATCCCAGCTACGAC
E6-A2(2).F	ATCTTGAATTCGCAGTAGTCCAGGGTTTCC
E6-A2(2).R	ATTAGCTCGAGACTGGAAAGACCGCGAAGAG
**RT-PCR**	
pcDNA.F	CACTGCTTACTGGCTTATCGAA
pcDNA.R	CTAGAAGGCACAGTCGAGGCT
Exon6.F	ATAATTCCCCCACCACCTCC
Exon7.R	AGGTGCTCACATTCCTTAAATT
Exon8.F	GAAATGCTGGCATAGAGCAGC
Exon8.R	ACTACAACACCCTTCTCACAG
